# Chronic Disease Management in Sub-Saharan Africa: Whose Business Is It?

**DOI:** 10.3390/ijerph6082258

**Published:** 2009-08-14

**Authors:** Alexander Bischoff, Tetanye Ekoe, Nicolas Perone, Slim Slama, Louis Loutan

**Affiliations:** 1 Institute of Nursing Science, Faculty of Medicine, University of Basel, Switzerland; 2 Department of Community Medicine and Primary Care, Division of International and Humanitarian Medicine, Geneva University Hospitals, Geneva, Switzerland; E-Mails: nicolas.perone@hcuge.ch (N.P.); slim.slama@hcuge.ch (S.S.); louis.loutan@hcuge.ch (L.L.); 3 Faculté de Médecine et des Sciences biomédicales, University of Yaoundé I, Yaoundé, Cameroon; E-Mail: tetanyeekoe@yahoo.fr (T.E.); 4 Faculty of Medicine, University of Geneva, Geneva, Switzerland

**Keywords:** nursing, chronic disease management, Sub-Saharan Africa, public health

## Abstract

Public health specialists and clinicians alike agree that Humanity faces a global pandemic of chronic diseases in the 21^st^ century. In this article we discuss the implications of this pandemic on another global issue, the health workforce. Because both issues are particularly acute in Sub-Saharan Africa (SSA), we will focus on this region and use Cameroon as a case in point. We first gauge the epidemic of chronic conditions in SSA. We then discuss the implications of chronic conditions for the reshaping of health systems and the health workforce. We conclude by making a strong case for the building up and strengthening the health workforce, insisting on the crucial role of nurses, their training, and involvement in chronic disease management.

## 1. Public Health and Chronic Diseases

Public health specialists and clinicians alike agree that Humanity faces in the 21^st^ century a global pandemic of chronic diseases [1]. There is an undeniable shift in health problems, away from acute conditions to chronic health problems, that has far-reaching implications and poses predictable and significant threats to all countries, especially to their health systems and the workforces. The pandemic of chronic conditions is caused, on the one hand, by non-communicable diseases such as diabetes [2], cardiovascular diseases [3] and mental disorders [4] and, on the other hand, by long-term communicable diseases such as HIV/AIDS and tuberculosis [5].

Recently, many studies have been conducted on the rise of the non-communicable diseases in high-income and low-income countries. However, these studies often dichotomize communicable and non-communicable diseases, with the communicable disease advocates and the non-communicable disease advocates, each on their respective side, claiming that ‘their’ epidemic has been neglected [6]. In such discussions it is easily overlooked that it is the chronicity that places such a heavy burden on the health services and the health workforce.

Since the 2006 World Health report, there has been an increasing awareness of the crucial role of the health workforce. However, this and related issues have been discussed mainly in terms of general workforce shortages, and less in terms of the need for more health professionals (and highly educated health professionals, we should add) combined with the increase of healthcare-demanding chronic conditions (communicable or non-communicable).

Therefore, in this article we discuss the implications of the one issue—the epidemic of chronic conditions—on the other, i.e., the workforce. Because both issues are particularly acute in Sub-Saharan Africa, we will focus on this region and use Cameroon as a case in point.

We first attempt to gauge the epidemic of chronic conditions in Sub-Saharan Africa (SSA). We provide up-to-date epidemiological information by compiling data that measure the extent of the chronic conditions epidemic in SSA, focusing on four among the most important: two communicable diseases—HIV/AIDS and tuberculosis, and two non-communicable diseases—diabetes and hypertension. We then discuss the implications of chronic conditions for the reshaping of health systems and the health workforce. We conclude by making a strong case for the building up and strengthening the health workforce, insisting on the crucial role of nurses, their training, and involvement in chronic disease management (CDM).

## 2. The Pandemic of Chronic Diseases

Chronic conditions are estimated to cause more than 60% of all deaths worldwide (up to 35 million in 2005). More than 80% of these deaths occur in the developing world [7]. These regions, including Sub-Saharan Africa (SSA), suffer not only from an increase in non-communicable diseases, but also– and still–from communicable diseases. Some authors use the term ‘double burden’ to describe the combined communicable and non-communicable disease epidemic [8]. The rise of chronic conditions places new long-term demands on health care systems for several reasons [5]. Because chronic conditions require ongoing management over a period of years or a lifetime, health systems must plan for increasing burden and complexity of care. Also, chronic conditions include acute episodes in a chronic trajectory; this combination challenges the functioning of current health systems, which are geared towards the treatment of acute disease [9], and tests our abilities to organize systems to meet pressing demands. Finally, and most importantly, chronic conditions engender increasingly serious economic and social consequences in all regions and threaten healthcare resources in every country [10]. If not successfully managed, they will become the most expensive problem faced by our health systems. In this respect, they pose a threat to all countries from both health and economic standpoints. This situation becomes particularly acute in Sub-Saharan Africa. Not only does this region have the highest burden of disease, it also has the weakest health systems and workforces, as well as the lowest per capita income. It has been shown that the poorest people have the highest risk of developing a chronic condition, and that they are the least able to cope with a chronic disease [7,11].

## 3. Chronic Conditions in Sub-Saharan Africa

It is only recently that non-communicable and communicable diseases have been viewed as sub-categories of chronic conditions [5]. This is contrary to the traditional biomedical view that focuses on causes. Setel *et al.* argue that classification of disease burdens based on aetiology and health transition theory is of limited utility to health planners in low-income countries. Instead, they propose ‘a broad care needs framework to complement the broad cause grouping’ [12]. This care needs approach, based on acuteness-chronicity, demonstrates to health planners the need to provide ‘services for the management of chronic conditions in health systems in Africa’. The care needs approach has vital consequences not only for the organizations of routine health services but also for the health workforce and the training thereof, especially the nursing workforce. Nurses are particularly affected because of the fact that they represent the main bulk of the health workforce. This issue will be dealt with in more detail further on, because epidemiological changes and challenges are discussed by and large among bio-medical researchers that may mention ‘non-physicians’, but do not fully recognize the essential role of the nursing sector.

According to UNAIDS [13], about 40 million people are currently infected with HIV, of whom about 64% are in SSA. In SSA, the overall HIV prevalence has decreased slightly over the last few years and is now around 6%, but the absolute number of people living with HIV is still on the increase. This is due to both the overall rise of the SSA population and to people surviving thanks to ART (antiretroviral therapy) treatment. A recent UNAIDS report classifies the HIV prevalence in Africa in three categories: <1% (very few countries, e.g., Senegal); 1%–5% (the group comprising the largest number of SSA countries); 5%–10% (including Cameroon, among other countries); and >10%, the group with a large number of countries in Southern Africa (including Botswana, with the highest prevalence at 38.5%).

According to The World Bank (Online Atlas of the Millennium Development Goals), the figure for Goal 6 of the MDG regarding Cameroon, for instance, is as follows: the prevalence of HIV, as expressed in percentage of the population aged 15 to 49, is 5.2% in Cameroon (for comparison: South Africa 19%, Switzerland 0.4%). According to the recent WHO Report on Tuberculosis, there were 8.8 million new cases worldwide in 2005; in SSA, these figures amounted to 2.5 million people [14]. The incidence of tuberculosis in Cameroon is 179 per 100,000 per year (for comparison: South Africa 718, Switzerland 7). Tuberculosis is thus the other major chronic condition that adds to the global epidemic of chronic disease in SSA. By just looking at the epidemiology of these two diseases, we can see that the need for chronic disease management (CDM) in African countries is enormous. Also, one chronic condition cannot be dealt with without taking other chronic conditions into account: HIV prevalence in adult incidence TB cases is as high as 26% in Cameroon (in South Africa the TB in HIV prevalence is 58%; in Switzerland 6%) [14]. The associations between these two diseases are becoming so close, that any selective (vertical) programme dealing with only one disease is becoming virtually useless.

Cameroon, to use an example for other Sub-Saharan countries, is crumbling under the ‘double burden’ [8] of communicable and non-communicable diseases. As examples of important non-communicable diseases, we present epidemiological data on diabetes and hypertension. Worldwide, 3.2 million deaths per year are attributable to diabetes, compared with 3.1 million deaths for AIDS. In SSA, the number of people with diabetes is approximately 7.2 million [15]. While in SSA infectious diseases are still major contributors to mortality and morbidity, non-communicable diseases add to the epidemic of chronic conditions, with diabetes being number two just after cardio-vascular diseases [2].

To make matters worse, the prevalence of diabetes will increase with improved access to ART [16], because ART is associated with a four-fold increase in the risk of diabetes [17]. In Cameroon, baseline data suggest that diabetes prevalence has increased more than ten-fold over ten years [16,18]. The prevalence estimate of diabetes mellitus in Cameroon was 0.8% in 2003 (South-Africa 3.4%) [19]. Hypertension affects approximately 59 million people worldwide [20]. In SSA, between 10 and 20 million people suffer from hypertension [21]. Also, hypertension in SSA is universally under-diagnosed or inadequately treated. Furthermore, hypertension frequently co-exists with other non-communicable risk factors, such as diabetes (21:254). Studies on hypertension in Cameroon show that the prevalence of high blood pressure >160/95 mmHg among males in urban areas is 16.4% and among females 12.1% (in rural areas 5.4% and 5.9% respectively). For comparison, in South Africa, the prevalence of hypertension among African males is 12.9% and among females 15.7% [21].

Mortality data are the most easily available epidemiological data. In terms of burden on health systems, however, these data are not optimal. In order to quantify the actual burden of disease comprehensively, WHO has developed the DALY measure, an approach that results in a single summary measure of morbidity, disability, and mortality, the so-called disability-adjusted life year (DALY) [22]. ‘One DALY can be thought of as 1 lost year of healthy life, and the burden of disease as a measurement of the gap between the present health of a population and an ideal situation in which everyone in the population lives into old age in full health’ (7:1930). In Table 1 we compile DALY data for the four diseases: HIV/AIDS, Tuberculosis, Diabetes, and Hypertension (excerpted from WHO statistics ‘WHOSIS’ [23]). The table shows that the DALYs of the four respective diseases account for 21% of the total DALYs in SSA, and even 29% in Cameroon. Also HIV/AIDS DALYs represent 76% of the respective DALYs worldwide. These epidemiological data show that the ‘big four chronic conditions’ with their long-term demands pose an enormous challenge for health services and health professionals in SSA [24].

## 4. Challenges of Chronic Diseases for Healthcare Delivery

The challenges for healthcare delivery are at least four-fold: first is the double burden— communicable diseases due the AIDS pandemic and the still high prevalence and virulence of TB and non-communicable diseases such as diabetes and cardio-vascular disease [25]; second is the ongoing burden that chronic conditions impose on health systems; third is the burden of multiple chronic conditions—studies show that more and more people have more than one chronic conditions [26,27]; and fourth is the burden on the primary healthcare (PHC) framework. This last burden has thus far not received the attention it deserves. The Alma Ata Declaration—30 years ago—shifted the focus away from the ‘mono-culture’ of curative services towards comprehensive health improving measures. Treating diseases was always a facet of PHC. However, common diseases were thought to be easy-to-treat ailments and not the highly complex treatment regimens generally needed when for chronic conditions.

The rise of chronic conditions is therefore a serious threat to the PHC achievements with its focus on a grassroots, bottom-up, decentralized, district-based approach. Chronic conditions are so complex to treat, that almost any programme addressing chronic conditions is likely to be a specialist one. The demands on funding and logistics are huge, including the needs to have strong links between the peripheral (district) level and the central level where the resources are concentrated. While the classical PHC approach is horizontal, chronic conditions programmes tend to be vertical. This in turn re-ignites the ‘selective PHC versus comprehensive PCH’ debate, which was thought to be overcome [28]. The 1978 Alma Ata Declaration on primary healthcare (WHO and UNICEF 1978) focused on low-cost, potentially high-impact interventions, both medical and social, at both the primary and community levels. Over time, the extent of resources and capacities needed to implement such comprehensive activities led to the emergence of the ‘selective primary healthcare’ concept [29]. Sepulveda argues that the dilemma between vertical (selective) programmes with proactive, disease-specific interventions on a massive scale and horizontal (comprehensive) programmes referring to more integrated, demand-driven, resource-sharing health services is a false one, proposing that ‘both need to coexist in what could be called a diagonal approach—that is, the proactive, supply-driven provision of a set of highly cost-effective interventions on a large scale that bridges health clinics and homes’ [30].

## 5. Nurses’ Response to the Chronic Conditions Epidemic

To face the epidemic of chronic conditions, the health system and its workforce have to change radically. A paradigm shift is needed: it is only very recently that public health and epidemiology are shifting ‘away from the health concerns of the 20^th^ century when acute diseases, quite easily treatable, and mainly by doctors, were the primary focus in every country’ [31] towards chronic conditions that currently account for more than half of the global disease burden and that will be the primary challenge for 21^st^-century healthcare systems. This applies equally to countries and health systems in the North and in the South.

There are several approaches to manage the epidemic of chronic diseases, most of which are based on the chronic care model. While the chronic care model was developed in the US and put into practice mainly by doctors, Bodenheimer, a physician, contends, by stating that ‘the healthcare literature and the experience of many efforts to improve chronic care indicate that nurses, not doctors, are the key to implementing the chronic care model in a patient centred care team’ (32:612). Various studies show the crucial role of nurses in chronic disease management (CDM). For example, patients attending a diabetes clinic with a nurse had lower mortality and a lower incidence of adverse clinical events than those patients receiving usual care [33]. A review found that nurses in primary care ‘can even replace physicians in delivering many aspects of diabetes care, if detailed management protocols are available, or if they receive training’ [34]. In terms of cost-effectiveness of CDM, nurse-led programmes may yield high quality outcomes [35–37], often similar or even better compared to physician-based CDM [38–40]. The bulk of this research has been undertaken in countries in high-income countries, but there is also evidence suggesting good cost-effectiveness of nurses involved in CDM in countries in low-income countries [41–43].

The shift from acute disease to chronic illness therefore requires also a paradigm shift in the health workforce [44]. Since this turn towards chronic diseases implies such a radical shift in the organization of a health system like the one in Cameroon, other findings in the literature around CDM, that were formulated for the context of high-income countries, are equally important: ‘The essence of the chronic care model is the interaction between an informed, activated patient and a prepared, proactive practice team. Indeed, such a team is nearly always needed to enable patients to become adequately informed and activated’ (32:612). Many of the positive outcomes seen in planned care visits with nurses may be due to better communication with patients. Nurses appear to be particularly apt as team players and are able to establish a–perhaps more–beneficial interaction with patients (than doctors). Therefore, nurses should be well prepared to assume the epidemiologic challenge of addressing the worldwide chronic conditions epidemic. This is important, because traditionally nurses in SSA play an essential role in ensuring primary care coverage, especially at the district and peripheral levels, where there are hardly any primary care doctors involved.

## 6. The Chronic Conditions Epidemic and the Consequent Workforce Requirements

If nurses are the ‘key to implementing the chronic care model’ [32], where are these nurses? And how do the numbers (of nurses or lacking nurses) translate in policy development concerning the preparation of the nurse workforce in SSA? In this section we provide some recent workforce-related figures on SSA in general, and on Cameroon in particular. Africa carries 25% of the world’s disease burden yet has only 3% of the world’s health workers (and 1% of the world’s economic resources to meet that challenge) [45]. A survey conducted in 2005 in the WHO African Region [46] showed that there were 621,164 nurses and 150,459 doctors and a group of 666,314 “other” health workers that included managers, administrators, dentists, laboratory workers and others. As in other world regions [47], nurses are by far the most numerous group of health workers, counting at least four times the amount of doctors. Data on the existing health workforce in SSA point to an acute shortage. The WHO estimates of health personnel per 100,000 population in SSA [48] are as follows.

In Cameroon the figure for physicians is 7.4 (Ghana 9.0, South Africa 25.1, the African average being 25.1), while the figure for nursing is 36.7, a low figure, even when compared to other African countries (Ghana 64.0, South Africa 140.0, the African average being 93.5). According to Mills, in 2004, there were 3,124 physicians and 25,997 nurses in Cameroon [49]. In Cameroon, there was an additional complication: a policy of non-recruitment of health personnel in the public sector for more than 15 years [50]. This was one of the consequences of a government reform in the early 1980s as part of the structural adjustment programme, administered by the World Bank. Also, training for nurses was suspended for several years and schools closed. Not surprisingly, in 1999, jobs in the public sector were about 80% unfilled and Cameroon had a truly de-motivated national health workforce’ [50]. To make matters worse, with the increase of chronic conditions all over SSA there is an urgent need for more health professionals. However, there are even less health professionals due to health workforce migration. In 2002, 52% of health workers intended to migrate and to leave Cameroon (Ghana 62%, Senegal 38%, and South Africa 58%) [51]. It is difficult to get realistic estimates of how many nurses actually leave the country and therefore how much workforce the respective country is losing. Liese [50] provides an appalling figure for South Africa: the ratios of physicians and nurses per 100,000 population dropped by 55% and 70%, respectively, between 1996 and 2001.

It is difficult to calculate what the necessary number of nurses would be to face the nurse workforce shortage in conjunction with the chronic conditions epidemic challenge. Recently, Kurowski and colleagues have calculated the needs in terms of health professionals to cope with primary care programmes in Tanzania [52]. Another study estimated the number of additional health workers needed to face the HIV epidemic in SSA (that is ‘only’ HIV/AIDS, not to mention the other workconsuming chronic conditions [53]): the number of health workers in SSA has to triple each year if every person with HIV or AIDS is to get anti-retroviral therapy. It has been calculated that provision of antiretrovirals to 1000 patients requires one or two doctors and seven nurses, ‘a reality that many resource-poor countries consistently fall short of’ [54].

To summarize, there are several factors for the workforce crisis in Africa and the nursing shortage [48,51]: HIV/AIDS prevalence among nurses [55]; internal migration of nurses (from rural to urban areas); international migration of nurses, largely to developed countries [49,56]; limited career and professional opportunities [57]; health sector reform which focuses on cost containment only, and budget cuts for nurses’ salari1es (which are already very low compared to other socio-professional categories [47]); a limited supply of new nurses; poor human resource management systems, which reflect the inefficiencies of the public sector health systems in recruitment, deployment, retention and motivation [58]; and limited career and professional opportunities resulting in frustration and consideration of health professions as undesirable [51].

There is thus a great and urgent need to produce more health workers able to cope with the chronic conditions epidemic. While under the buzzword of task shifting [59], the involvement of community health workers has been advocated, there will always be an urgent need to produce more nurses. Nurses will have to take on supervision tasks, not the least because task shifting implies also the shifting of doctors’ tasks to nurses.

## 7. Implementing Chronic Disease Management: The Case of Cameroon

This last section sketches out a plan to prepare the necessary nursing workforce capable to manage chronic conditions. The training of nurses with the necessary skills in CDM should be implemented at both service delivery level (continuing education for nurses working in primary care settings) and at the basic nurse education level for nurse students. CDM was developed in the USA (and other high-income countries) [60]. The WHO, realizing that chronic conditions would be one of the main challenges for health systems not only in high-income, but also in low-income countries, developed the new Innovative Care for Chronic Conditions building blocks model. This model draws heavily on the chronic care model and its offspring [61–63]; its ingredients can be summarized with the following C’s: continuity–ongoing relationship between provider and patient [24]; coordination–collaboration in care team and involving additional (lay) health workers [64]; communication [65]–the patient’s understanding of the treatment plan leading to successful self-management and decision making [61]; cooperation–with patients as partners [61]; consultations–that are centred on the patients [66]; and community-linked–so that prevention and control of diseases can be linked to community-participation [63].

The keys to success in CDM are the same for all chronic diseases: HIV/AIDS, tuberculosis, diabetes and hypertension. So far, examples exist where these CDM concepts have been implemented [67], but very few were carried out in SSA. The results of one of them indicate that appropriate CDM can be achieved in the SSA context by optimizing human resources and involving nurse-led clinics [42]. We conclude this paper by describing four examples related to CDM in Cameroon.

First, a cross-sectional survey examined the relation between provider-patient interaction in a rural health district in Cameroon [68]. More than 75% of the providers were nurses. The study identified important obstacles to implement disease management, in particular the fact that patients were more likely to understand their illness when the nurses provided more explanations about the disease and the respective illness concepts. Also, most patients who understood the diagnostic said they agreed with the nurses concerning the provided diagnosis. The study adds to the body of knowledge that is need to improve disease management programmes.

Second, in a continuing education programme, an intervention aimed at improving communication was tested [69,70]. The results, based on a randomised trial and with the so-called RIAS (Roter Interaction Analysis Scale) [71], showed that the communication behaviour of the intervention group in the consultation improved (as compared to the control group) in the areas such as: paraphrasing, checking and back-channelling responses; encouraging patients to speak; verifying whether the patient understood what had been explained; reassuring and showing empathy; showing less disapproval; providing health education messages; and providing understandable explanations. Also, patients in the intervention group: asked more questions; talked more easily about their life-style; and discussed HIV/AIDS more openly. In a subsequent phase, a training programme for district nurses in the health centres of one large district is currently being implemented, the main topic being the management of chronic conditions, including diabetes, hypertension and HIV/AIDS.

Third, a programme was developed with the aim to strengthen HIV prevention of mother-to-child transmission. The programme trained health professionals, most of them nurses, in a number of health districts in rural Cameroon [72]. The sequence of its elements included the following: nurses received trained (2-day workshops on prevention of mother-to-child transmission) on how to deal with HIV/AIDS prevention; nurses’ knowledge was tested by means of a questionnaire survey; nurses were given equipment, drugs and algorithm-guidelines. In a follow-up survey after seven months, the nurses were again tested by means of the same questionnaire. The study showed that the introduction of a HIV/AIDS prevention of mother-to-child transmission programme is feasible, but to ensure sustainability additional supervision is needed to increase the impact.

Finally, a new nurse education programme [73] has recently been introduced at university level, in accordance with a curricula review of courses offered at the Faculty of Medicine and Biomedical Sciences, University of Yaoundé. This review had been triggered, on the one hand, by the wish to build the three-tiered nursing education with Bachelor, Masters and Doctorate, and by the changing epidemiological and public health challenges. Importantly, the focus of the new curriculum is on the management of chronic diseases, since it became clear that, with the advent of large scale anti-retroviral therapies [53], nurses will belong to the main players and may even be the leaders at district level. They will have to be able to work on their own, and, with the fast development of tele-communication systems, under the supervision of the medical district officers in charge. A large part of the course deals with the management of chronic conditions at district level, including the communicable diseases such as HIV/AIDS and TB, and the non-communicable diseases such as diabetes and hypertension. Capacity building in CDM allows for integrating the respective concepts into the framework of PHC (Primary Health Care), which, among other things, decentralizes and ‘horizontalizes’ healthcare, so that the district level (tertiary level) is the fundamental part of the health system. The new curriculum is in line with recent WHO reviews and policies [74], and includes the five core competencies [31]: patient-centred care; partnering; quality improvement; information and communication technology; and public health perspective.

This article attempted to show that the way to tackle the epidemic of chronic conditions and its consequent challenges for health systems in care delivery in SSA is to strengthen nurses in their capacity to manage chronic conditions. Innovative nurse training programmes with emphasis on chronic disease management are likely to be the most promising strategies.

## Figures and Tables

**Table 1.** Overview of burden of disease measures, expressed in DALYs (2002) of the ‘big four’ chronic conditions (HIV/AIDS, TB, Diabetes, Hypertension).

	**Global DALYs**	**SSA DALYs**	**Cameroon DALYs**
HIV/AIDS	84,457,784	63,962,104	1,434
Tuberculosis	34,735,908	9,266,350	680
Diabetes	16,194,381	1,114,952	107
Hypertension	7,646,994	586,467	15
ALL CAUSES	1,490,125,643	361,376,478	7,615
*Proportion of the four diseases among all causes (%)*	*9.5%*	*20.7%*	*29.3%*
